# Effect of the Frequency of Self-Monitoring Blood Glucose in Patients with Type 2 Diabetes Treated with Oral Antidiabetic Drugs—A Multi-Centre, Randomized Controlled Trial

**DOI:** 10.1371/journal.pone.0003087

**Published:** 2008-08-28

**Authors:** Werner A. Scherbaum, Christian Ohmann, Heinz-Harald Abholz, Nico Dragano, Mark Lankisch

**Affiliations:** 1 Department of Endocrinology, Diabetes and Rheumatology, WHO Collaborating Centre for Diabetes, European Training Centre in Endocrinology and Metabolism, University Hospital Düsseldorf, Düsseldorf, Germany; 2 German Diabetes Clinic, German Diabetes Centre at the Heinrich-Heine-University Düsseldorf, Düsseldorf, Germany; 3 Coordination Centre for Clinical Trials, University Hospital Düsseldorf, Düsseldorf, Germany; 4 Department of General Medicine, University Hospital Düsseldorf, Düsseldorf, Germany; 5 Department of Medical Sociology, University Hospital Düsseldorf, Düsseldorf, Germany; University of Bremen, Germany

## Abstract

**Objective:**

Recommendations on the frequency of self-monitoring of blood glucose (SMBG) vary widely among physicians treating patients with type 2 diabetes (T2D). Aim of this study was to investigate two testing regimen of SMBG in patients with stable metabolic control.

**Research Design and Methods:**

Patients with T2D treated with oral antidiabetic drugs were randomized to two groups: either one SMBG (low) or four SMBG (high) per week. Subjects were followed up after 3, 6 and 12 months. Primary outcome parameter was the change in HbA1c between baseline and 6 months. Primary outcome criterion was tested by a one-sided t- test for non- inferiority. Secondary outcome parameters were safety, compliance and HbA1c at 3 and 12 months.

**Results:**

There were no differences in the 202 subjects for demographic and sociodemographic parameters and drug treatment. HbA_1_c (%) at baseline was similar in both groups (7.2±1.4 *vs.* 7.2±1.0). Non- inferiority was demonstrated for the low group (p = 0.0022) with a difference from baseline to 6 months of 0.24 in the low and of 0.16 in the high group. Compliance with the testing regimen was 82–90% in both groups. There were no statistical significant differences for compliance, HbA_1_c at 3 and 12 months and serious adverse events (SAE).

**Conclusion:**

One SMBG per week is as sufficient and safe as four SMBG per week to maintain HbA_1_c in non-insulin treated T2D close to metabolic target. The results of this study are in contrast to current international consensus guidelines.

**Trial Registration:**

Controlled-Trials.com ISRCTN79164268

## Introduction

Self-monitoring blood glucose (SMBG) improves glycaemic control in patients with type 1 diabetes [1] and possibly also in insulin-treated type 2 diabetes (T2D) [2], especially when treated with multiple insulin injections per day [3]. However, the value and frequency of SMBG in non-insulin-treated patients with T2D is a matter of controversy.

A consensus opinion among a group of experts from the UK suggested that patients with T2D using oral antidiabetic drugs (OAD) should monitor their blood glucose at least once daily, varying the time of testing between fasting, preprandial and postprandial levels during the day [4]. A global consensus conference on SMBG recommended eleven measurements a week in these patients [5] and another recent consensus conference noted that patents with T2D on OAD may use SMBG but specific recommendations with respect to frequency were not made [6].

A cross-sectional and longitudinal study of patients with T2D in Australia showed that HbA1c was not significantly different between SMBG users and nonusers, either overall or within diabetes treatment groups such as diet, OAD or insulin, with or without OAD [7]. Although such observational data can be useful in determining the effect of an intervention [8], conclusive evidence of this assumption is not available from randomized controlled trials.

A recent study reported on the effect of a more and less intensive diabetes education combined with recommendations on the frequency of SMBG in patients with T2D [9]. They found that a more intensive education did not result in an improved HbA1c (%) compared to standard information and care.

Our study was designed to strictly test the effect of the frequency of SMBG without changing the other diabetes management.

Our hypothesis was that one SMBG measurement a week in a stable phase of metabolic control close to glycemic target is no less effective than more measurements a week with regard to metabolic control, hypoglycaemia and/or hyperglycemias, or adverse events (AE).

## Methods

The protocol for this trial and supporting CONSORT checklist are available as supporting information; see Checklist S1, Protocol S1 and Protocol S2.

### Study design

A prospective multi-centre randomized controlled trial was performed evaluating non-inferiority of low vs. high frequencies of SMBG with respect to metabolic control T2D. The study was approved by the ethical committees of all six institutions participating, and all patients provided their written informed consent.

### Study population

Patients with T2D treated with one or more OAD (not combined with insulin therapy and stable oral medication for the last three months) aged between 35 and 80 years were enrolled. Patients with following conditions were excluded: type 1 diabetes, advanced renal insufficiency (creatinine level at ≥2.5 mg/dl), at least two episodes of hypoglycaemia requiring external support within the previous three months, one or more severe metabolic events (hypoglycemic shock, hyperosmolar coma, inpatient stay due to severe hyperglycaemic events) within the past three months, pregnancy, severe impairment of vision or communication problems due to language.

All patients had received a structured education on diabetes mellitus and instructions on SMBG in practice before the start of the study, and were not specifically re-educated.

Originally, it was planned to establish a second trial arm with insulin treated patients, comparing one measurement a week (low group) with 11 measurements a week (high group). Due to poor recruitment this arm was terminated early by the trial steering committee. The reasons for poor recruitment were mainly changes in diabetes treatment in Germany due to a move from pre-mixed insulin to a different type of insulin treatment.

### Study intervention

Patients were randomly assigned to two strategies of SMBG. Low group: SMBG with one measurement a week and additional measurement in the event of suspected hypoglycaemia or severe hyperglycaemia or high group: four measurements a week on Tuesdays, Thursdays and one day of the weekend before dinner and one additional measurement before lunch, and also additional measurement in the event of suspected hypoglycaemia or severe hyperglycaemia. Hypoglycaemia was defined as an SMBG<3.2 mMol/L (<60 mg/dl). Severe hypoglycaemia was defined as any hypoglycaemia with the need for assistance by another person.

The randomization list was generated and centrally applied with concealment by the Coordination Centre for Clinical Trials with the use of randomly selected block sizes of 4, 6 and 8 patients stratified according to centre.

In our case-management approach, all patients were asked to report back to their physician in the event of inappropriate diabetes control according to the targets set between the patient and his or her doctor.

### Evaluation and follow-up

Potential study patients were screened according to inclusion and exclusion criteria. After randomization, patients were instructed on the strategy of SMBG to be applied for a period of 6 months (formal instruction, written instruction, patient diary, delivery of equipment for measuring blood glucose). Patients were evaluated at the initial visit and after 3 and 6 months by clinical investigation, assessment of (serious) adverse events, quality of life (at baseline and at 6 months only), compliance with intervention, change in diabetes treatment, socioeconomic effects and measurement of HbA1c. After 12 months only HbA1c was assessed. In addition, blood glucose measurements were documented by the patient in a diary, and the data from the first week each month were used to assess intervention compliance from the patient's viewpoint. Compliance with treatment was defined as follows: maximal deviation of one measurement from the allocated frequency (e.g. 3 to 5 in the high group, 0 to 2 in the low group).

### Trial outcomes

The primary outcome criterion was the change in HbA1c levels between baseline and six months after enrolment. Secondary outcomes were number and type of hypoglycaemic and hyperglycaemic events, quality of life, compliance and satisfaction with interventions, socioeconomic effects and HbA1c after 3 and 12 months. Data on quality of life, satisfaction with intervention and on the socioeconomic background will not be presented in this paper.

### Statistical analysis

The study was designed to test non- inferiority of the low group compared to the high group. Sample-size calculation was based on data from the German Diabetes Centre (590 patients, mean value (standard deviation) of HbA1c: 9.95±1.83), a one-sided *t*-test for non-inferiority assuming an acceptable deviation of 0.5% for the difference in HbA1c levels between baseline and 6 months, statistical power of 80% and an alpha error of 5% leading to an estimated sample size of 167 patients per group. Taking drop-outs into consideration (e.g. informed consent withdrawn, lost to follow-up), sample size was calculated at 400, stratified into two strata: patients with oral medication (n = 200) and patients with insulin treatment (n = 200). All analyses were performed according to intention to treat and included all randomized patients excluding the drop-outs. The primary outcome criterion was tested by a one-sided *t*-test for non-inferiority. The alternative hypothesis was stated as follows: the difference in the low group is equal to or better than in the high group, taking a deviation of 0.5% into consideration [10]. Comparability between study groups and secondary endpoints were investigated by chi-square test for qualitative and *t*-test, Mann- Whitney- test for quantitative data.

## Results

**Figure 1:**
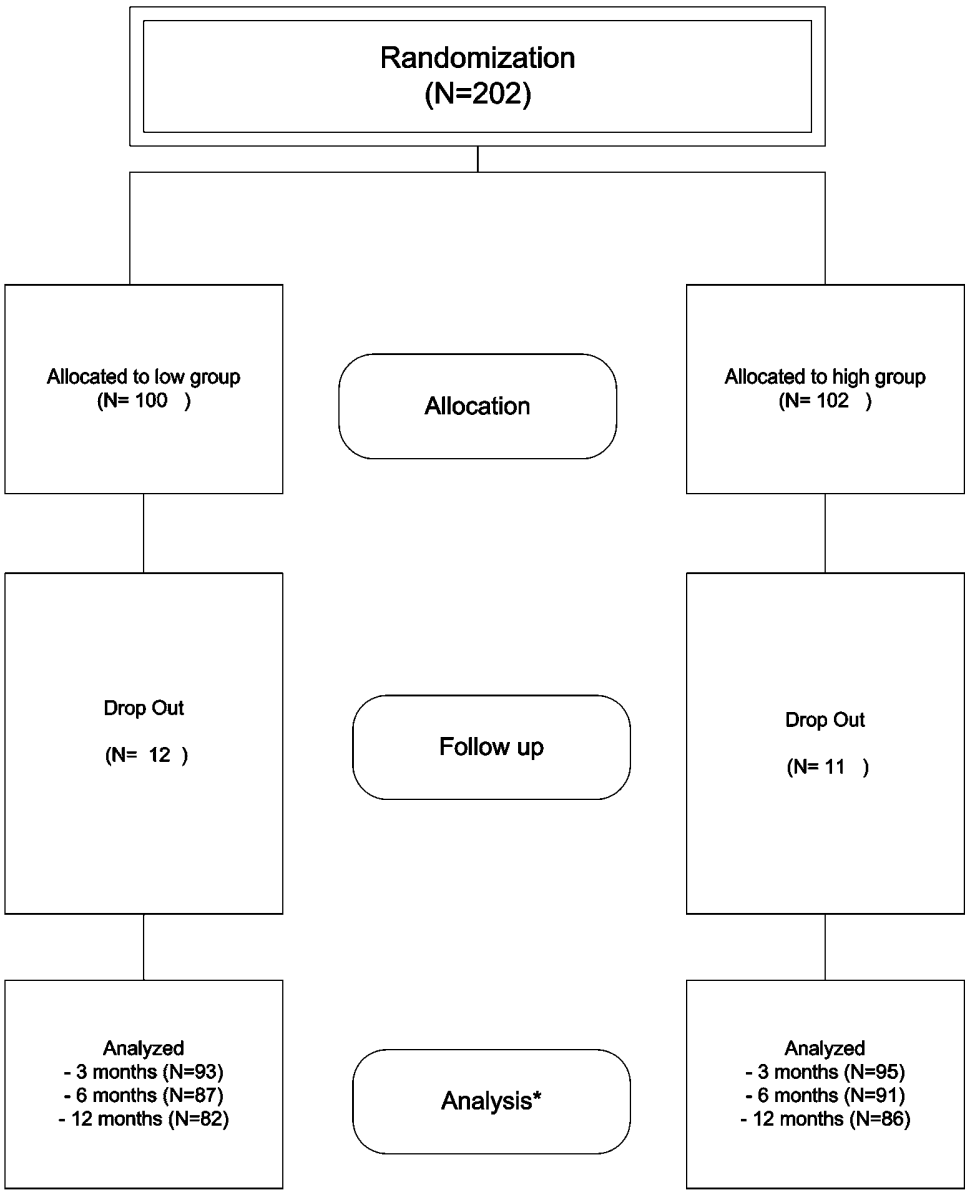
Flow of participants in the trial (according to CONSORT statement). * with respect to primary outcome criterion HbA1c.

A total of 202 patients from six centres underwent randomization in the oral group between Dec 2003 and Oct 2005; 100 were randomly assigned to the low and 102 to the high group. There were 12 drop-outs in the low and 11 drop-outs in the high group (Fig. 1). The baseline characteristics in the two groups were similar (Table **1**, **2**). Mean diabetes duration was 7.8±6.4 years in the low and 8.2±6.5 in the high group.

**Table 1:** Characteristics of the patients.

Characteristic	Intervention		P Value
	Low	High	
	(N = 100)	(N = 102)	
**Age** - yr (mean±SD)	61.7±11.7	61.0±9.0	0.68
**Sex**			
- male	60/100 (60%)	65/102 (64%)	0.66
- female	40/100 (40%)	37/102 (36%)	
**Duration of diabetes** - yr	7.8±6.4	8.2±6.5	0.71
**History**			
- coronary heart disease	19/100 (19%)	16/101 (16%)	0.58
- myocardial infarction	8/100 (8%)	11/102 (11%)	0.63
- stroke	2/100 (2%)	2/102 (2%)	1.00
- peripheral arterial disease (PAD)	13/100 (13%)	14/102 (14%)	1.00
- other serious disease	34/100 (34%)	28/102 (28%)	0.36
- diabetic nephropathy	2/100 (2%)	3/102 (3%)	1.00
- diabetic neuropathy	25/100 (25%)	21/102 (21%)	0.50
- diabetic retinopathy	2/100 (2%)	4/102 (4%)	0.68
- relevant hypoglycemia*	3/100 (3%)	4/102 (4%)	1.00
- relevant hyperglycemia*	6/100 (6%)	3/102 (3%)	0.32
**HbA1c** - %	7.2±1.4	7.2±1.0	0.53
**Nationality**			
- german	84/98 (86%)	89/102 (87%)	0.84
- other	14/98 (14%)	13/102 (13%)	
**Graduation**			
- none	3/96 (3%)	1/102 (1%)	
- elementary school	46/96 (48%)	44/102 (43%)	
- secondary school	12/96 (12%)	17/102 (17%)	
- advanced technical college qualification	11/96 (11%)	15/102 (15%)	
- higher education entrance qualification	22/96 (23%)	20/102 (20%)	
- other	2/96 (2%)	5/102 (5%)	
**Marital status**			
- single	4/98 (4%)	6/102 (6%)	
- married	72/98 (73%)	70/102 (69%)	0.77
- living separate	4/98 (4%)	7/102 (7%)	
- divorced	11/98 (11%)	9/102 (9%)	
- widowed	7/98 (7%)	10/102 (10%)	
**Employment Situation**			
- employed	16/98 (16%)	26/101 (26%)	
- part time employed	6/98 (6%)	3/101 (3%)	
- old-age pension	42/98 (43%)	41/101 (40%)	
- early retired	17/98 (17%)	13/101 (13%)	0.48
- unemployed/short-time work	8/98 (8%)	10/101 (10%)	
- housewife	7/98 (7%)	4/101 (4%)	
- other	2/98 (2%)	4/101 (4%)	

* within previous three months.

**Table 2:** Characteristics of the patients.

Characteristic	Intervention		P Value
	Low	High	
	(N = 100)*	(N = 102)	
**Medication**			
- Acarbose	1/99 (1%)	3/102 (3%)	0.62
- Glibenclamide	23/99 (23%)	19/102 (19%)	0.42
- Glimepirid	25/99 (25%)	29/102 (28%)	0.61
- Gliquidon	0/99 (0%)	1/102 (1%)	1.00
- Glyburide	0/99 (0%)	1/102 (1%)	1.00
- Metformin	70/99 (71%)	76/102 (75%)	0.55
- Nateglinide	2/99 (2%)	1/102 (1%)	0.62
- Pioglitazone	3/99 (3%)	2/102 (2%)	0.68
- Repaglinide	7/99 (7%)	10/102 (10%)	0.49
- Rosiglitazone	9/99 (9%)	5/102 (5%)	0.24

* one patient with missing data.

### Compliance and adverse effects

Compliance with the assigned frequency of SMBG was measured from the doctor's and the patient's viewpoint (Table **3**). Compliance in the low group assessed by the doctor was 61% at 3 months and 73% at 6 months. In the high group, compliance to the SMBG regimen assigned was 77% at 3 months and 83% at 6 months. Analysis of each patient's diary revealed compliance in 82–83% (0 to 3 months) and 85–88% (4 to 6 months) in the low group and 87–90% and 84–88% in the high group. A statistical significant difference was observed according to doctor's assessment at 3 months (p<0.03).

**Table 3:** Assessment of compliance.

Assessment of compliance*	Intervention		P Value
	Low	High	
	(N = 100)	(N = 102)	
**Investigator**			
- 3 months	57/92 (61%)	71/92 (77%)	0.03
- 6 months	62/85 (73%)	75/91 (83%)	0.14
**Patients diary** *			
3 months			
- first week in month 1	72/88 (82%)	83/95 (87%)	0.31
- first week in month 2	73/88 (83%)	83/95 (87%)	0.41
- first week in month 3	72/88 (82%)	85/95 (90%)	0.30
6 months			
- first week in month 4	73/86 (85%)	77/92 (84%)	0.84
- first week in month 5	73/84 (87%)	80/91 (88%)	1.00
- first week in month 6	73/83 (88%)	77/92 (84%)	0.82

* at most one measurement less or more than defined.

Clinicians were asked to record all AE's and SAE's according to Good Clinical Practice (GCP). The number and type of SAE and AE was similar between the groups except for hypoglecaemia, which was increased in the high group (Table **4**). Twenty patients from the low group and fifteen in the high group showed SAE. No hypoglycaemic shock or hyperosmolar coma occurred. Inpatient hospital treatment was necessary in 19 patients in the low group and 14 in the high group. There was one death in the low and one death in the high group. Both deaths were unrelated to the study intervention (pancreatic cancer, heart failure).

**Table 4:** Outcome and Safety.

Outcome and Safety	Intervention		P Value
	Low	High	
	(N = 100)	(N = 102)	
**HbA1c** - %			
**- baseline**			
mean±SD† (valid observations)	7.2±1.4 (100)	7.2±1.0 (102)	0.92*
**- 3 months**			
mean±SD (valid observations)	6.9±1.2 (93)	6.9±0.7 (95)	0.66*
**- 6 months**			
mean±SD (valid observations)	6.9±1.2 (87)	7.0±0.8 (91)	0.53*
**- 12 months**			
mean±SD (valid observations)	6.9±1.0 (82)	7.1±1.0 (86)	0.10*
**Health care utilization**			
**between 0–3 months**			
- physician visit	65/89 (73%)	68/92 (74%)	1.00
- inpatient stay**	13/90 (15%)	7/92 (8%)	0.16
- incapacity to week	11/86 (13%)	10/90 (11%)	0.81
**between 4–6 months**			
- physician visit	67/85 (79%)	61/91 (67%)	0.09
- inpatient stay**	11/87 (13%)	12/91 (13%)	1.00
- incapacity to week	13/83 (16%)	13/85 (15%)	1.00
**Change from oral hypoglycaemic**			
**agents to insulin**	5/100 (5%)	9/102 (9%)	0.41
- between 0–3 months	4/100 (4%)	6/102 (6%)	0.75
- between 4–6 months	1/100 (1%)	3/102 (3%)	0.62
**Adverse Events**			
- relevant hypoglycemia			
- one event	1/100 (1%)	9/102 (9%)	0.02
- several events	4/100 (4%)	9/102 (9%)	0.25
- relevant hyperglycemia	1/100 (1%)	1/102 (1%)	1.00
- deterioration neuropathy	0/100 (0%)	0/102 (0%)	1.00
- deterioration retinopathy	0/100 (0%)	0/102 (0%)	1.00
- deterioration nephropathy	0/100 (0%)	0/102 (0%)	1.00
- multiple	6/100 (6%)	4/102 (4%)	0.54
- other	7/100 (7%)	3/102 (3%)	0.21
**Serious Adverse Events**			
- hypoglycaemic shock	0/100 (0%)	0/102 (0%)	1.00
- hyperosmolar coma	0/100 (0%)	0/102 (0%)	1.00
- other	20/100 (20%)	15/102 (15%)	0.58
- inpatient stay	19/100 (19%)	14/102 (14%)	0.57
- death	1/100 (1%)	1/102 (1%)	1.00

* t- Test.

† SD = Standard Deviation.

** Hospital, treatment at a health resort, rehabilitation, others.

### Outcome

HbA1c at baseline was similar in both groups (7.2±1.4 *vs.* 7.2±1.0) (Table **4**). The primary outcome criterion (difference of HbA1c value at baseline and after 6 months) was assessed in 178 patients (low group: n = 87, high group: n = 91); non-inferiority was demonstrated for the low group (p = 0.0022) with a mean difference of 0.24 in the low and of 0.16 in the high group. In addition, no statistically significant differences in HbA1c values at 3 and 12 months were observed (Table **4**). There were no statistically significant differences between the groups with respect to health care utilization (physician visits, inpatient treatment, or days off work) and changes in diabetes treatment (Table **4**).

## Discussion

To our knowledge, this is the first randomized, controlled prospective multi-centre trial on the frequency of SMBG in patients with T2D treated with OAD with a strict focus on the specific impact of the frequency of SMBG. Patients in this cohort were in a stable phase of T2D close to glycemic target and they had been on the same anti-diabetic treatment for at least three months. In a large observational cohort study (ROSSO), we recently demonstrated that patients with T2D who apply SMBG had better outcomes in terms of morbidity and mortality than patients that did not [11]. However, the database from this cross-sectional study did not allow to provide any answer to the optimal frequency of SMBG in T2D.

The need for SMBG in patients with T2D has been discussed controversially. A recently published review of randomized controlled trials came to the conclusion that SMBG is likely to be helpful in non-insulin-treated patients with T2D, but the methodological quality of the studies analyzed was considered to be low [12]. Welschen and colleagues defined nine quality criteria that should ideally be met in this type of study. In their review on the literature concerning SMBG, they found 1572 citations, but only six studies were considered as appropriate for further analysis [13]–[18]. However, the design of these six studies only met 5, 5, 5, 5, 6, or 7 out of nine methodological quality criteria. Drawbacks were, among others, failure to conceal treatment allocation (all 6), blinding of the outcome assessor to the intervention [13]–[16], [18], unaccounted or unacceptable withdrawal or drop-out rate [18], or failure to include an intention-to-treat analysis [13]–[16]. The conclusions drawn from these studies are limited. All of the above quality criteria except blinding of the outcome assessor were met in our study.

We considered one SMBG measurement as clinically important in order to allow the patient to detect unexpected metabolic deterioration between doctor's visits. Four SMBG measurements a week in our high group is by far less than the eleven measurements a week that were suggested by a global consensus panel [5]. The decision to suggest a lower number of SMBG in patients with T2D treated with OAD and an HbA1c close to target was based on our own experience in outpatient care of patients with T2D. There is also a substantial disparity between the actual and recommended frequency of SMBG testing. A previous analysis of data from German health care providers demonstrated that patients with T2D on insulin therapy or on OAD have a test strip use of 1.8 and 0.8 strips a week, respectively [19]. It is also common experience that patients with HbA1c close to target may not show much change, and differences between groups would most probably be minimal [20].

Our primary endpoint of change in HbA1c demonstrated non-inferiority in the low compared to the high group. One concern we had with respect to the performance of the study was that patients assigned to the low group would measure more frequently and patients in the high group would measure less frequently. However, most patients in the two groups kept a high level of compliance to their assigned testing regimen as assessed by patient diary.

As mentioned above, our study was designed to strictly evaluate the effect of the frequency of SMBG on metabolic control without mixing this tool with different education procedures or intensities in diabetes management. In the study by Farmer et al. [9] it appeared that only about half of the patients in the more intensive group followed the recommendations regarding SMBG and the other performed less than 2 SMBG per week. It may be critisized that the failure to show an effect on HbA1c in this study is due to a lack of adherence to the study regimen. In our trial, however, the adherence in both groups, i.e. low and high frequency of SMBG, was as high as 82–90%.

No differences were observed with respect to AE's and SAE's, except for hypoglycaemia, which occurred more often in the high group probably due to the higher frequency of measurements.

Although our data show no advantage of more than one SMBG measurement a week in non-insulin treated T2D patients it is important to emphasize that these results only refer to patients with stable metabolic control and without any necessity of changing antidiabetic medication. The conclusions drawn from our study do not refer to patients with newly diagnosed T2D or patients with intercurrent diseases disturbing metabolic control. Although prospective controlled trials are still missing to this point a change in the treatment regimen and frequency of SMBG measurements may be necessary in such patients [5].

### Conclusion

This study shows that in patients with T2D treated with OAD one SMBG measurement a week is not associated with any deterioration in metabolic control (HbA1c) or therapeutic safety as compared to four measurements a week. Under study conditions compliance for a low frequency of SMBG was surprisingly high. Implementation of the study results in clinical routine would result in considerable cost-savings and it would be very important for the sake of type 2 diabetic patients not to have to measure SMBG more than once weekly.

## Supporting Information

Protocol S1 Trial Protocol, in German.
(0.51 MB DOC)

Protocol S2 Trial Protocol, in English.
(0.02 MB PDF)

Checklist S1 CONSORT Checklist.
(0.06 MB DOC)
