# Supplementary material for: Effect of the Frequency of Self-Monitoring Blood Glucose in Patients with Type 2 Diabetes Treated with Oral Antidiabetic Drugs—A Multi-Centre, Randomized Controlled Trial
Source: PLoS One. 2008 Aug 28;3(8):e3087. doi: 10.1371/journal.pone.0003087 (PMC2518209; doi:10.1371/journal.pone.0003087)
Supplement: Protocol S1 — Trial Protocol, in German. (0.51 MB DOC) [file pone.0003087.s001.doc]

**Untersuchung zur Frequenz notwendiger Blutzuckerselbstmessungen bei Typ 2- Diabetes**

###### **Eine prospektive, kontrollierte, randomisierte, multizentrische Studie**

Förderung im Rahmen des BMBF - Programms zur "Stärkung und Förderung der Versorgungsforschung"

**Version:** 1.3

**Datum:** 15.10.04

**Status:**  Version nach Amendment

**Studienleitung:**

Leiter klinischer Prüfung:

Prof. Dr. med. W. A. Scherbaum, Deutsches Diabetes Forschungsinstitut an der Heinrich-Heine-Universität Düsseldorf, Deutsche Diabetes Klinik

Prof. Dr. med. H.-H. Abholz, Direktor der Abteilung für Allgemeinmedizin, Universitätsklinikum Düsseldorf

**Biometrie:**

Prof. Dr. rer. nat. C. Ohmann, Koordinierungszentrum für Klinische Studien an der Heinrich-Heine-Universität

**Ethik:**

Ethikkommission der HHU Düsseldorf/ Ethikkommission der Ärztekammer Nordrhein

**Studienzentrale:**

Koordinierungszentrum für Klinische Studien (KKS) an der Heinrich-Heine-Universität

**Sponsor:**

Bundesministerium für Bildung und Forschung (BMBF)

**Förderer:**

Roche Diagnostics GmbH

**1. Allgemeines:**

**Studienleitung:**

Leiter klinischer Prüfung:

Prof. Dr. med. W. A. Scherbaum, Deutsches Diabetes Forschungsinstitut an der Heinrich-Heine-Universität Düsseldorf, Deutsche Diabetes Klinik, Auf'm Hennekamp 65, 40225 Düsseldorf

Tel.: 0211 / 33 82 200, Telefax: 0211 / 33 69 103, e-mail: scherbaum@ddfi.uni-duesseldorf.de

Prof. Dr. med. H.-H. Abholz, Abteilung für Allgemeinmedizin, Universitätsklinikum Düsseldorf, Postfach 10 10 07, 40001 Düsseldorf, Telefon: 0211 / 81 17771

Telefax: 0211 / 81 18755, e-mail: abholz@med.uni-duesseldorf.de

**Biometrie:**

Prof. Dr. rer. nat. C. Ohmann, Koordinierungszentrum für Klinische Studien an der Heinrich-Heine-Universität, Moorenstr. 5, 40225 Düsseldorf, Telefon: 02211 / 811 9701

Telefax: 0211 / 8119702, e-mail: ohmannch@uni-duesseldorf.de

**Ethik:**

Ethikkommission der HHU Düsseldorf/ Ethikkommission der Ärztekammer Nordrhein

**Studienzentrale:**

Koordinierungszentrum für Klinische Studien (KKS) an der Heinrich-Heine-Universität, Moorenstr. 5, 40225 Düsseldorf, Janett Schindler, Telefon: 0211/81 19710, Telefax: 0211/81 19705, e-mail: janett.schindler@uni-duesseldorf.de

**Sponsor:**

Bundesministerium für Bildung und Forschung (BMBF)

Roche Diagnostics GmbH

**Beteiligte Wissenschaftler:**

Prof. Dr. P. Oberender, Ordinarius für Volkswirtschaftslehre und Wirtschaftstheorie. Forschungsstelle für Sozialrecht und Gesundheitsökonomie, Universität Bayreuth,

95440 Bayreuth, Tel.: 0921 / 552881, Telefax: 0921/ 55 28 86, e-mail: peter.oberender@uni-bayreuth.de

Prof. Dr. rer. nat. G. Giani, Direktor Abteilung Biometrie und Epidemiologie Auf'm Hennekamp 65, 40225 Düsseldorf, Telefon: 0211 / 3382 259, Telefax: 0211 / 3382 677, e-mail: [giani@ddfi.uni-duesseldorf.de](mailto:giani@ddfi.uni-duesseldorf.de)

Prof. Dr. J. Siegrist, Direktor Institut für Medizinische Soziologie und Zusatzstudiengang "Gesundheitswissenschaften und Sozialmedizin", Heinrich-Heine-Universität Düsseldorf

Medizinische Einrichtungen, Universitätsstr.1, Gebäude 23.02, Ebene 03, 40225 Düsseldorf, Telefon: 0211 / 81 14360/61, Telefax: 0211 81 12390, email: [siegrist@uni-duesseldorf.de](mailto:siegrist@uni-duesseldorf.de)

Dr. med. M. Lankisch, Deutsches Diabetes Forschungsinstitut an der Heinrich-Heine-Universität Düsseldorf, Deutsche Diabetes Klinik, Auf'm Hennekamp 65, 40225 Düsseldorf

Tel.: 0211 / 33 82 200, Telefax: 0211 / 33 69 103, email: lankisch@uni-duesseldorf.de

Frau S.N. Emeklibas, Koordinierungszentrum für Klinische Studien an der HHU Düsseldorf, Telefon: 0211-81-19712, email: SelimeN.Emeklibas@uni-duesseldorf.de

Herr N. Dragano, M.A. Institut für Medizinische Soziologie, Heinrich-Heine-Universität Düsseldorf,

Universitätsstraße 1, 40225 Düsseldorf, Telefon: 0211 / 8114360

Telefax: 0211/ 8114366 e-mail: dragano@uni-duesseldorf.de

Dr. med. S. Wilm Abteilung für Allgemeinmedizin, Universitätsklinikum Düsseldorf, Postfach 10 10 07, 40001 Düsseldorf, Telefon: 0211 / 81 17771

Dr. med. S. Labrenz, Deutsches Diabetes Zentrum an der Heinrich-Heine-Universität Düsseldorf, DDZ , Auf'm Hennekamp 65, 40225 Düsseldorf, Telefon: 0211/33 82593, e-mail: labrenz@ddfi.uni-duesseldorf.de

## Vertraulichkeitshinweis

Der Inhalt von Protokoll und Prüfbogen ist vertraulich zu behandeln und darf (ohne Zustimmung der Protokollleitung) weder mündlich noch schriftlich an Unbeteiligte weitergegeben werden

## Beteiligte Personen, Institutionen und Gremien

Teilnehmende Prüfärzte/ Prüfzentren:

Prof. Dr. med. W. A. Scherbaum, Deutsches Diabetes Forschungsinstitut an der Heinrich-Heine-Universität Düsseldorf, Deutsche Diabetes Klinik, Auf'm Hennekamp 65, 40225 Düsseldorf

Tel.: 0211 / 33 82 200, Telefax: 0211 / 33 69 103, e-mail: scherbaum@ddfi.uni-duesseldorf.de

Prof. Dr. med. M. Pfohl, Medizinische Klinik I des Evangelischen Krankenhaus Bethesda zu Duisburg gGmbH, Heerstraße 219 , 47053 Duisburg-Hochfeld

Tel.: 0203/ 60 08 1351, Telefax.: 0203/ 60 08 2359

Dr. Cüppers, Diabetes-Zentrum Rheinland, St. Josef Krankenhaus, Robert Koch Straße 16, 42781 Haan

Tel.: 02129/ 92 90

Prof. Dr. Mies, Chefarzt der inneren Abteilung des St. Antonius – Krankenhaus, Schillerstrasse 23, 50941 Köln

Tel.: 0221/ 37 93 0

Teilnehmende Hausärzte:

Dr. A. Dost, Dr. Th. Dost, Neutor 28-32, 46325 Borken,

Tel.: 02861/ 64 534

G. Wallenfang, Kirchhellener Straße 255 A, 46240 Bottrop,

Tel.: 0204/ 97 52 77

Dr, B. Lobscheid, Kirchplatz 1, 40670 Meerbusch,

Tel.: 02159/ 24 02

Weitere Hausärzte werden nachgemeldet.

# Unterschriftenliste

Studienleitung:

Leiter klinischer Prüfung

Prof. Dr. med. W. A. Scherbaum, Deutsches Diabetes Forschungsinstitut an der Heinrich-Heine-Universität Düsseldorf, Deutsche Diabetes Klinik, Auf'm Hennekamp 65, 40225 Düsseldorf

Tel.: 0211 / 33 82 200, Telefax: 0211 / 33 69 103, e-mail: scherbaum@ddfi.uni-duesseldorf.de

**Datum/Unterschrift**

Prof. Dr. med. H.-H. Abholz, Abteilung für Allgemeinmedizin, Universitätsklinikum Düsseldorf, Postfach 10 10 07, 40001 Düsseldorf, Telefon: 0211 / 81 17771

Telefax: 0211 / 81 18755, e-mail: abholz@med.uni-duesseldorf.de

**Datum/Unterschrift**

Biometrie:

Prof. Dr. rer. nat. C. Ohmann, Koordinierungszentrum für Klinische Studien an der Heinrich-Heine-Universität, Moorenstr. 5, 40225 Düsseldorf, Telefon: 02211 / 811 9701

Telefax: 0211 / 8119702, e-mail: ohmannch@uni-duesseldorf.de

**Datum/Unterschrift**

# Inhaltsverzeichnis

**1. Allgemeines**

**2. Zusammenfassung**

**3. Flowchart**

**4. Einführung/ Hintergrund/ Fragestellung**

4.1. Stand der Forschung und Versorgungssituation im jeweiligen Handlungsbereich im internationalen Vergleich

**4.2. Eigene Vorarbeiten**

**4.3. Gesundheitspolitische Relevanz des Themas, Bedeutung für die Regelversorgung der Patienten und für die gesetzliche Krankenversicherung**

**5. Studienziele**

**5.1. Zielkriterien**

**5.1.1. Haupt-Zielkriterium**

**5.1.2. Nebenzielkriterien**

**5.1.2.1. Zahl und Ausmaß hypoglykämischer und hyperglykämischer Entgleisungen**

**im Studienzeitraum**

**5.1.2.2. Krankheitsspezifische Lebensqualität, Therapiecompliance und Therapiezufriedenheit**

**5.1.2.3. Pharmako-ökonomische Berechnungen**

**5.1.2.4. HbA1c-Wert nach 12 Monaten**

**6. Studiendesign**

# 7. Auswahl der Patienten/ Auswahlkriterien

**7.1. Setting**

**7.2. Therapiegruppen (Strata**)

**7.2.1.** **Insulin-behandelte Typ 2-Diabetiker**

**7.2.2.** **Mit oralen Antidiabetika behandelte Diabetiker**

#### 7.3. Ein und Ausschlusskriterien

**7.3.1. Einschlusskriterien**

**7.3.2. Ausschlusskriterien**

**8. Aufnahme/ Registrierung und Randomisation**

**9. Studienablauf**

**9.1. Therapiegruppen**

**9.1.1. Insulin-behandelte Typ 2-Diabetiker**

**9.1.2. Mit oralen Antidiabetika behandelte Diabetiker**

**10. Klinische Untersuchungen/ Statuserhebungen**

**11. Ablauf und Dauer der Studie**

### **12. Ermittlung der Wirksamkeit/ Erfassung der therapeutischen Effektivität**

**12.1. Vergleich der gesundheitsbezogenen Lebensqualität**

**12.2. Therapiezufriedenheit- und compliance**

**12.3. Gesundheitsökonomische Betrachtung**

**12.3.1. Folgekosten**

**12.3.2. Direkte Kosten der Blutzuckermessung**

**12.3.3. Erfassung möglicher indirekter Kosten**

#### 13. Ermittlung der Sicherheit

**13.1. Erfassung und Bewertung von unerwünschten Ereignissen**

**13.1.1. Unerwünschte Ereignisse**

**13.1.2. Schwerwiegende unerwünschte Ereignisse**

**13.2. Dokumentation und Bewertung unerwünschter Ereignisse**

**14. Dauer der Studie / Studienabbruch**

**14.1. Reguläres Studienende**

**14.2. Vorzeitiges Studienende / Abbruch der gesamten Studie**

**15. Statistische Methoden**

**15.1. Grundlage der Berechnung**

**15.2. Studienhypothesen**

**15.3. Auswertungsfolge**

**15.4. Intention-to-treat / Per-protocol Analyse**

**15.5. Fallzahlkalkulation**

**15.6. Randomisierung**

#### 16. Datenmanagement

**16.1. Patientenidentifikationsliste**

**16.2. Datenerhebung / Dokumentationsbögen**

**16.3. Datenverarbeitung**

**16.4. Aufbewahrung der Studienunterlagen**

**17. Qualitätssicherung**

**17.1. Standardisierung**

**17.2. Kontrolle des Studienablaufs und der Datenqualität**

#### 18. Ethische Grundlagen

1. **Literatur**

**2. Zusammenfassung**:

| Zusammenfassung | Prospektive, randomisierte, kontrollierte Multizenter-Studie |
| --- | --- |
| Endpunkte | Primärer Endpunkt:  Differenz des HbA1c-Wertes 6 Monate nach Beginn der Studie zu dem Vorstudienwert  Sekundäre Endpunkte:  1. Hypoglykaemie mit Fremdhilfe und hyperosmolares Koma  2. Lebensqualität, Therapiecompliance und Therapiezufriedenheit  3. Pharmakoökonomische Betrachtung  4. HbA1c-Wert nach 12 Monaten |
| Einschlusskriterien | - Patienten, die 2 x täglich ein Mischinsulin bekommen, d. h. eine Mischung von schnell wirksamem und mittel oder lang wirksamem Insulin - Patienten, die mit einem oralen Antidiabetikum oder einer Kombinationstherapie ohne Insulin eingestellt sind - 35- 80 Jahre - Schriftliche Einverständniserklärung zur Studienteilnahme (informed consent) - Typ 2- Diabetes mellitus |
| Ausschlusskriterien | - Typ 1- Diabetes mellitus - Fortgeschrittene Niereninsuffizienz (bekannter Kreatinin (> 2.5 mg/dl), - ≥ 2 Hypoglykaemien mit notwendiger Fremdhilfe in den letzten 3 Monaten - ≥ 1 schwere Stoffwechselentgleisung (hypoglykämischer Schock **/** hyperosmolares Koma stationäre Aufenthalte wegen schwerer hyperglykämischer Entgleisungen) in den letzten 3 Monaten - Schwangerschaft - Schwere Sehstörungen - Sprachverständigungsschwierigkeiten - Konsumierende Erkrankungen - Ambulante Pflege/ Pflegedienst |
| Intervention | Insulintherapiegruppe  I. Maximalempfehlung: jeden Tag nüchtern, jeden zweiten Tag (Dienstag, Donnerstag und ein Wochenendtag) vor dem Abendessen BZ Kontrolle, zusätzlich 1 x wöchtliche BZ-Messung vor dem Mittagessen  II. Minimalempfehlung: 1 x pro Woche Nüchternblutzucker und bei V.a. Hypoglykämie oder ausgeprägte Hyperglykämie  Orale Diabetikergruppe  I. Maximalempfehlung: jeden zweiten Tag (Dienstag, Donnerstag und ein Wochenendtag) Nüchternblutzucker, 1 x pro Woche BZ Kontrolle vor dem Abendessen.  II. Minimalempfehlung: 1 Nüchternblutzucker pro Woche. |
| Fallzahl | Einseitiger T-Test auf Äquivalenz bei Intention- to- treat- Analyse. Bei einem klinisch relevanten Unterschied des HbA1c Wertes von 0,5% wird bei α = 0,05 und einer Power von 80%, eine Fallzahl von 167 Personen pro Studienarm kalkuliert. Einschließlich der Drop-outs ergibt sich eine Zahl von 200 Patienten pro Studienarm (orale und Insulintherapie), bei zwei Armen insgesamt 400 Patienten. |

**3. Flowchart**


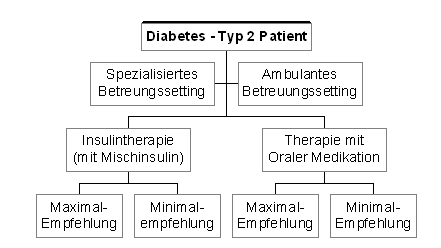


**4. Einführung/ Hintergrund/ Fragestellung**

4.1. Stand der Forschung und Versorgungssituation im jeweiligen Handlungsbereich im internationalen Vergleich

Derzeit gibt es in Deutschland etwa 5 Millionen bekannte Typ 2- Diabetiker. Davon werden nahezu 1 Million mit Insulin behandelt. Etwa 50% dieser Insulintherapien erfolgen mit einer Gabe von 2 x tägl. Mischinsulin. Etwa 2 Millionen Diabetiker werden alleine mit oralen Antidiabetika behandelt. Die Blutglukoseselbstmessung ist ein wichtiger Bestandteil der Blutzuckereinstellung, insbesondere bei insulinbehandelten Diabetikern. Zwischen den Empfehlungen von Diabetologen und den in der Praxis durchgeführten Selbstkontrollen gibt es jedoch große Unterschiede. Diese liegen in einer Spanne zwischen mehrmals täglichen Blutzuckerselbstmessungen und auf der anderen Seite Messung von nicht einem Blutzucker pro Quartal. International gibt es dazu bisher überwiegend nur Beobachtungsstudien (1) (2-4) sowie sehr kleine prospektive randomisierte Studien (5), die alle nahe legen, dass die Zahl von Blutzucker-Selbstmessungen beim Typ 2-Diabetes auch bei einer konventionellen Insulintherapie nicht mit der Güte der Blutzuckereinstellung (gemessen am HbA1c-Wert und an der Frequenz von Hypoglykämien) korreliert. Diese sehr kleinen Studien, sowie nicht randomisierte und nicht prospektive Beobachtungsstudien legen nahe, dass die Zahl von Blutzuckerselbstmessungen beim Typ 2- Diabetes auch bei einer konventionellen Insulintherapie nicht mit der Güte der Blutzuckereinstellung (gemessen am HbA1c und an Hypoglykämien) korreliert (5;6).

**4.2. Eigene Vorarbeiten**

Die Antragsteller verfügen über umfangreiche Erfahrungen in der Durchführung klinischer Studien, speziell auf dem Gebiet der Versorgungsforschung. Professor Scherbaum ist spezialisiert auf dem Gebiet des Diabetes mellitus und zentrumsbasierter klinischer Untersuchungen; Herr Prof. Abholz betreibt ein Netzwerk von Lehrpraxen, mit denen er bereits zahlreiche, hausarztbasierte Untersuchungen durchgeführt hat. Herr Prof. Ohmann ist als Leiter des Koordinierungszentrums für Klinische Studien mit der gesamten Logistik und Infrastruktur Klinischer Studien bestens vertraut. Herr Dragano aus der Abteilung von Herrn Prof. Siegrist (Direktor Institut für Medizinische Soziologie und Zusatzstudiengang "Gesundheitswissenschaften und Sozialmedizin", Heinrich-Heine-Universität Düsseldorf) bringt die sozialmedizinischen Aspekte mit in die Studie ein. Die gesundheitsökonomischen Analysen werden insbesondere von Herrn Prof. Oberender erarbeitet, der auf dem Gebiet gesundheitsökonomischer Analysen in klinischen Studien über einschlägige Erfahrungen verfügt. Herr Prof. Giani ist als Leiter der Abteilung Biometrie und Epidemiologie des DDFI und in der biometrischen Analyse von Studiendaten speziell ausgewiesen.

**4.3. Gesundheitspolitische Relevanz des Themas, Bedeutung für die Regelversorgung der Patienten und für die gesetzliche Krankenversicherung**

Mit dieser prospektiven kontrollierten randomisierten multizentrischen Studie kann erstmals die sehr wichtige Frage geklärt werden, ob die von Fachdiabetologen empfohlene Zahl von Blutzucker-Selbstmessungen bei Patienten sinnvoll ist. Damit wird erstmals eine Evidenzbasis für entsprechende Empfehlungen für die Praxis geschaffen. Die mit dieser Studie gewonnenen Daten sind essentiell für die Regelversorgung der Patienten. Gerade bei der Blutzucker-Selbstmessung kann bisher durch die ungenügende Datenlage noch nicht sicher und evidenzbasiert über Unter-, Über- und Fehlversorgung entschieden werden, was sowohl für die Ärzte und Leistungserbringer, aber auch für die Patienten entscheidend ist. Wir nehmen an, dass man für den Patienten einen Großteil der Belästigung durch die Selbstverletzungen im Rahmen der Blutzuckermessungen ersparen und gleichzeitig unnötige Kosten für die Krankenkassen einsparen könnte, ohne die Versorgungsqualität dieser Menschen zu verschlechtern. Es gibt sogar Hinweise darauf, dass diese Verminderung der Messfrequenz die Lebensqualität der Patienten erheblich verbessern könnte (1). Kalkuliert man pro Messung Kosten von 0,58 € [Minimalkalkulation, Range 0,58 € - 0,80 €] (7), so wäre bei einer Minimalmessung von 1 x pro Woche und einer Maximalmessung von 11 x pro Woche in der Insulingruppe bzw. von 4 x in der Gruppe mit oraler Antidiabetikatherapie eine Kostenersparnis von 5,80 € bzw. 1,74 € pro Woche zu erreichen. Rechnet man diese Zahl auf die oben angegebene Zahl von Typ 2- Diabetikern mit Insulintherapie in Deutschland hoch, so ergäbe sich eine Ersparnis von 150,8 Mio. € pro Jahr für die insulinbehandelten Patienten und 180,96. Mio € pro Jahr für die mit oralen Antidiabetika behandelten Patienten. Die Ergebnisse unserer Studie können ohne Verzug in die allgemeine Versorgungspraxis, insbesondere auch im hausärztlichen Bereich übertragen werden. Die eingesparten Gelder könnten besser für andere Elemente der Prophylaxe und Therapie diabetischer Folgeschäden verwendet werden.

**5.Studienziele**

Im Rahmen der geplanten Untersuchungen sollen anhand einer prospektiven kontrollierten randomisierten Studie erstmals valide Daten zur optimalen Frequenz von Blutzucker-Selbstmessungen in Abhängigkeit von der Behandlungsstrategie bei Menschen mit Typ 2-Diabetes erhoben werden.

Wir gehen von der Arbeitshypothese aus, dass eine Verringerung der Zahl von Blutzucker-Selbstmessungen weder zu einer klinisch relevanten Erhöhung des HbA1c-Wertes noch zu häufigeren Hypoglykämien führt, jedoch mit einer Verbesserung der Lebensqualität einhergeht. Eine Verringerung der Zahl von Blutzucker-Selbstmessungen würde zu erheblichen finanziellen Einsparungen führen.

**5.1. Zielkriterien**

**5.1.1. Haupt-Zielkriterium**

Das Haupt-Zielkriterium der Studie ist die Differenz des HbA1c-Wertes 6 Monate nach Beginn der Studie zu dem Wert bei Studienbeginn.

Häufige Blutzucker-Selbstmessungen sind nur dann gerechtfertigt, wenn sie zu einer Verbesserung der glykämischen Kontrolle führen, was mit einer Verminderung der mikrovaskulären Folgekrankheiten des Diabetes verbunden ist. Entsprechend internationalem Standard wird eine klinisch relevante Verbesserung der glykämischen Kontrolle bei Reduktion des HbA1c um mindestens 0,5 % angesehen.

**5.1.2. Nebenzielkriterien**

**5.1.2.1. Zahl und Ausmaß hypoglykämischer und hyperglykämischer Entgleisungen**

**im Studienzeitraum**

Solche Entgleisungen sind ein wichtiges Kriterium für die Qualität der Stoffwechseleinstellung; sie sollen durch die Blutzucker-Selbstmessungen und Möglichkeit zur Gegensteuerung durch den Patienten selbst mit Hilfe diätetischer Maßnahmen und Motivation der körperlichen Aktivität Anlass geben. Wir gehen jedoch davon aus, dass es zwischen beiden Gruppen keine Unterschiede geben wird. Die Einschätzung und Dokumentation der Episoden geschieht im Patiententagebuch, durch den Arzt und den DTSQ-Fragebogen (siehe Lebensqualität). Als signifikante Hypoglykämie wird eine Unterzuckerung mit Fremdhilfe gewertet, eine hyperklykämische Entgleisung als hyperosmolares Koma.

**5.1.2.2. Krankheitsspezifische Lebensqualität, Therapiecompliance und Therapiezufriedenheit**

Diese sind ein wichtiges patientenzentriertes Maß für die Qualität der Therapie beim Diabetes mellitus Die LQ wird mittels validierter Instrumente zu Beginn der Studie und 6 Monate danach erfasst und die Veränderungen in den Therapiegruppen werden verglichen. Der Fokus wird auf allgemeine Aspekte der gesundheitsbezogenen Lebensqualität gelegt, da hier LQ-Veränderungen zu erwarten sind. Mit der Messung der Therapiezufriedenheit und der Therapiecompliance sollen zwei direkte Maße für die Akzeptanz und objektive Bewertung der Therapien erfasst werden. Bei der Planung dieser Erfassung wurde Wert darauf gelegt, möglichst kurze und ökonomisch zu erfassende Instrumente zu verwenden.

**5.1.2.3. Pharmako-ökonomische Berechnungen**

Die regelmäßige hochfrequente Blutzucker-Selbstmessung ist mit erheblichen Kosten verbunden, die evtl. besser in die Frühdiagnose und Prävention von Langzeitfolgen des Diabetes investiert werden können. Sollte es sich herausstellen, dass häufige Blutzucker-Selbstmessungen nicht zu einer Verbesserung der Stoffwechseleinstellung führen, so kann dieses Geld eingespart werden. Im Rahmen der Studie werden

a) die Zahl der Arztbesuche bei allen Disziplinen, die den Diabetes betreffen b) die Zahl der Krankenhaustage und c) die Zahl der Tage mit Arbeitsunfähigkeit erhoben. Die Kosten für die Blutzuckermessung ergeben sich aus der Zahl der dokumentierten Blutzuckermessungen in den Patiententagebüchern.

**5.1.2.4. HbA1c-Wert nach 12 Monaten**

Um die Nachhaltigkeit der Interventionen zu überprüfen, soll zusätzlich der HbA1c-Wert nach 12 Monaten bestimmt werden.

**6. Studiendesign:**

In dieser Multicenterstudie auf Äquivalenz sollen insgesamt 400 Patienten/innen mit Typ 2-Diabetes in klinischen Studienzentren und dazugehörigen Hausarztpraxen untersucht werden. Die Untersuchung umfasst zum einen Patienten, die zweimal täglich ein Mischinsulin bekommen, und zum anderen solche, die ausschließlich mit oralen Antidiabetika eingestellt sind. Pro Studienarm werden je 100 Patienten den Blutzucker nach den Maximalempfehlungen und 100 nach den von uns vorgeschlagenen Minimalempfehlungen kontrollieren. Primärer Endpunkt ist die Differenz des HbA1c-Wertes 6 Monate nach Beginn der Studie zu dem Vorstudienwert. Als sekundäre Endpunkte werden Therapieakzeptanz, gesundheitsbezogene Lebensqualität, pharmako-ökonomische Berechnungen, HbA1c-Wert nach 12 Monaten und schwerwiegende unerwünschte Ereignisse (Hypoglykaemie mit Fremdhilfe und hyperosmolares Koma) herangezogen.

# 7. Auswahl der Patienten/ Auswahlkriterien

**7.1. Setting**

Es werden Patienten rekrutiert, die in Schulungs- und Behandlungszentren für Typ 2- Diabetiker (zertifiziert durch die Deutsche Diabetes Gesellschaft (DDG)) stationär behandelt oder im hausärztlichen Bereich betreut werden. Es handelt sich dabei um das Deutsche Diabetes Zentrum (DDZ) Düsseldorf, St. Antonius Krankenhaus Köln, Diabeteszentrum Rheinland Haan, Bethesdakrankenhaus Duisburg und angegliederte Arztpraxen. Die Follow-Up Visiten finden in den Zentren statt, aus dem der Patient rekrutiert wurde.

Die strickte Trennung der Settings in ein spezialisiertes und ein ambulantes Setting wurde aufgehoben, da sich die Funktion des Allgemeinmediziners in den letzten Jahren deutlich verändert hat. So wurde die Betreuung von Diabetikern auch in der allgemeinmedizinischen Praxis unter anderem durch das DMP Programm detaillierter und spezialisierter.

Der Allgemeinmediziner besitzt nicht mehr – wie zu Beginn der Studie angenommen – die Funktion des gate-Keepers im Gesundheitssystem sondern ist selbst durch umfangreiche Weiterbildungsmöglichkeiten (z. B. Diabetologe DDG) zum spezialisierten Diabeteszentrum geworden.

Auch unterscheiden sich die Schulungsmöglichkeiten für Patienten im DDZ und anderen Diabeteszentren nicht mehr in der Form, wie es zu Beginn der Studie angenommen wurde.

Patienten mit allen Schweregraden der Erkrankung werden durch die klinischen Zentren und die angegliederten Hausarztpraxen behandelt

**7.2. Therapiegruppen (Strata**)

Es werden zwei Gruppen von Patienten untersucht.

**7.2.1.** **Insulin-behandelte Typ 2-Diabetiker**

Patienten, die 2 x täglich ein Mischinsulin bekommen, d. h. eine Mischung von schnell wirksamem und lang wirksamem Insulin, dies seit mindestens 3 Monaten und

**7.2.2.** **Mit oralen Antidiabetika behandelte Diabetiker**

Patienten, die mit einem oralen Antidiabetikum oder einer Kombinationstherapie seit mindestens 3 Monaten ohne Insulin eingestellt sind.

#### 7.3. Ein und Ausschlusskriterien

**7.3.1. Einschlusskriterien:**

- Patienten, die 2 x täglich ein Mischinsulin bekommen, d. h. eine Mischung von schnell wirksamem und mittel oder lang wirksamem Insulin oder
- Patienten, die mit einem oralen Antidiabetikum oder einer Kombinationstherapie ohne Insulin eingestellt sind
- 35-80 Jahre
- Schriftliche Einverständniserklärung zur Studienteilnahme (informed consent)
- Typ 2-Diabetes mellitus

**7.3.2. Ausschlusskriterien:**

- Typ 1- Diabetes mellitus
- Fortgeschrittene Niereninsuffizienz (bekannter Kreatinin (> 2.5 mg/dl),
- ≥ 2 Hypoglykaemien mit notwendiger Fremdhilfe in den 3 Monaten
- ≥ 1 schwere Stoffwechselentgleisung (hypoglykämischer Schock / hyperosmolares Koma/ stationäre Aufenthalte wegen schwerer hyperglykämischer Entgleisungen) in den letzten 3 Monaten
- Konsumierende Erkrankungen
- Diabetesneueinstellung absehbar innerhalb der nächsten 4 Wochen
- Schwangerschaft
- Schwere Sehstörungen
- Sprachverständigungsschwierigkeiten
- Ambulante Pflege/ Pflegedienst

**8. Aufnahme/ Registrierung und Randomisation**

Die Studienpatienten werden nach schriftlicher Einwilligung und Stratifizierung nach insulinpflichtigen oder nicht insulinpflichtigen Diabetikern in eine von beiden Therapiegruppen (Maximalempfehlung, Minimalempfehlung) randomisiert. Die Randomisierung wird als zentrale Telefon- bzw. Computerrandomisierung über das KKS durchgeführt.

**9. Studienablauf**

**9.1. Therapiegruppen**

**9.1.1. Insulin-behandelte Typ 2-Diabetiker**

1.) Gruppe mit Maximalempfehlung der Blutzuckerselbstmessung wie folgt: täglich Blutzucker vor dem Frühstück, jeden zweiten Tag (Dienstag, Donnerstag und ein Wochenendtag) vor dem Abendessen (jeweils vor der Injektion) plus 1 x pro Woche eine Blutzuckermessung vor dem Mittagessen.

2.) Gruppe mit einer Minimalempfehlung: 1 x pro Woche Blutzucker vor dem Frühstück oder bei Symptomatik mit Hinweis auf Blutzuckerentgleisung (Hypoglykämie oder Hyperglykämie), insgesamt etwa 1 bis 2 Blutzuckerselbstmessungen pro Woche.

**9.1.2. Mit oralen Antidiabetika behandelte Diabetiker**

1.) Maximalempfehlung: jeden zweiten Tag (Dienstag, Donnerstag und ein Wochenendtag) Nüchternblutzucker sowie 1 x pro Woche Blutzucker vor dem Abendessen.

2.) Minimalempfehlung: 1 Nüchternblutzucker pro Woche.

**10. Klinische Untersuchungen/ Statuserhebungen**

Bei der Studie handelt es sich um eine pragmatische Studie bei Patienten mit stabiler Diabeteseinstellung, die die Therapiesteuerung durch Selbstmessungen untersuchen soll. Es erfolgen keinerlei Vorgaben zur Therapie der Patienten, damit die Studie den in der Praxis üblichen Ablauf optimal abbildet. Dies gilt auch für das Verhalten nach Hyper- oder Hypoglykämien. Therapeutische Maßnahmen werden - mit Begründung der Änderung - dokumentiert. Alle diabetesrelevanten unerwünschten klinischen Ereignisse sind zu dokumentieren. Sollten unerwünschte Ereignisse auftreten, so kann der Arzt auch angeben, warum keine Veränderungen vorgenommen wurden. Die Patienten haben zu standardisierten Zeitpunkten Angaben zum Thema Lebensqualität zu machen.

| Untersuchung | **Basisuntersuchung** | **Monat 3** | **Monat 6** | **Monat 12** |
| --- | --- | --- | --- | --- |
| Körperliche Untersuchung | X | X | X |  |
| Unerwünschte Ereignisse / Anamnese | X  (letzte 3 Monate) | X | X |  |
| HbA1c | X | X | X | X |
| Änderung der Diabetestherapie | X | X | X |  |
| Lebensqualitätserhebungen | X |  | X |  |
| Gesundheitsökonomische Erhebung | X | X | X |  |
| Fragebogen zur Therapiecompliance | X | X | X |  |

Ablauf der Erfassung

Der Ablauf der gesundheitsökonomischen Erhebung geschieht folgendermassen:

1. BZ Messung:

Die Kosten für jede BZ Messung sind bekannt und unabhängig vom Setting. Sie betragen ca. 0,7 € pro Messung. Mit Hilfe der Eintragungen in die Patiententagebücher lassen sich diese Kosten anhand der Zahl der Eintragungen sehr gut erheben und vergleichen. Die Eintragungen geschehen regelmäßig, die Tagebücher werden bei den Visiten eingesammelt, vor Ort photokopiert und anschließend dem Probanden wieder ausgehändigt. Diese Unterlagen werden am Ende der Studie ausgewertet.

Probanden, die schon ein Blutzuckermessgerät haben, können dieses weiterverwenden, alle anderen bekommen Messstreifen mit visueller Anzeige. Patienten die vorher noch nicht BZ gemessen haben werden dementsprechend geschult, als Stechhilfe werden Softclix zur Verfügung gestellt.

1. Ambulante Behandlung:

Es wird die Anzahl von Arztbesuchen abgeschätzt. Dazu wird die Zahl der ambulanten Arztbesuche vom Hausarzt oder im DDFI zu den Nachsorgeterminen oder bezüglich der letzten drei Monate vor Studieneinschluss dokumentiert. Die Erhebungen finden zu den Zeitpunkten 0, nach 3 Monaten und nach 6 Monaten statt.

1. Krankenhausaufenthalte:

Die Zahl der Krankenhausaufenthalte und die Zahl der im Krankenhaus verbrachten Tage werden vom Hausarzt oder im DDFI zu den Nachsorgeterminen oder bezüglich der letzten drei Monate vor Studieneinschluss dokumentiert. Die Erhebungen finden zu den Zeitpunkten 0, nach 3 Monaten und nach 6 Monaten statt.

1. Indirekte Kosten:

Die Zahl der Tage mit Arbeitsunfähigkeit wird vom Hausarzt oder im DDFI zu den Nachsorgeterminen oder bezüglich der letzten drei Monate vor Studieneinschluss dokumentiert. Die Erhebungen finden zu den Zeitpunkten 0, nach 3 Monaten und nach 6 Monaten statt.

| Erhebung | **Basis** | **mehrfach wöchentlich / täglich** | **Monat 3** | **Monat 6** | **Monat 12** |
| --- | --- | --- | --- | --- | --- |
| Blutzuckermessungen |  | X |  |  |  |
| HbA1c | X |  | X | X | X |
| Hyperglykaemien / Hypoglykaemien | X  (letzte 3 Monate) |  | X | X |  |
| Ambulante Behandlungen /  Krankenhausaufenthalte /Ausfalltage | X |  | X | X |  |

**11. Ablauf und Dauer der Studie**

In die Studie eingebunden werden vier Diabeteszentren in Nordrheinwestfalen: Das Deutsche Diabetes Zentrum (DDZ) Düsseldorf, das Diabetes Zentrum Rheinland Haan, das St. Antoniuskrankenhaus Köln und das Bethesda Krankenhaus Duisburg. Diese Zentren kooperieren mit einem Kreis niedergelassener Allgemeinmediziner und hausärztlich tätiger Internisten. Das DDZ arbeitet im Rahmen der Studien mit Forschungspraxen der Abteilung für Allgemeinmedizin an der Heinrich Heine Universität zusammen. Weitere Praxen werden sich im Rahmen eines Ärztenetzwerks für klinische Studien an dieser Untersuchung beteiligen.

Das Bethesda Krankenhaus Duisburg unter der Leitung von Herrn Professor Pfohl arbeitet mit niedergelassenen Kollegen im Rahmen eines Qualitätszirkels für Diabetes mellitus zusammen. Vergleichbare Strukturen finden sich auch im Diabetes Zentrum Rheinland unter der Leitung von Professor Cüppers und im St. Antonius Krankenhaus Köln unter der Leitung von Herrn Professor Mies.

Die Patienten werden entweder in den Praxen im Rahmen des DMP-Programmes erfasst oder im Rahmen einer Behandlung in einem der klinischen Diabeteszentren in die Studie eingeschlossen und dort auch weiterhin betreut. Es ist geplant pro Zentrum 100 Patienten zu rekrutieren wobei die Verteilung zwischen zentrumsbetreuten Patienten und hausarztbetreuten Patienten etwa 50:50 sein sollte. Abweichungen dieser vorgegebenen Verteilung werden aufgrund der regionalen Unterschiede in der Therapie des Diabetes mellitus Typ 2 möglich sein. So werden nach den bisherigen Erfahrungen in den klinischen Zentren der Studie in den letzten Monaten in den klinischen Zentren nur sehr wenige Patienten mit oraler antidiabetischer Therapie vorstellig, sodass der Großteil der geplanten 200 Patienten mit oraler Therapie eher über die angegliederten Hausarztpraxen rekrutiert wird.

Die Datenerfassung erfolgt mit Hilfe von CRF´s (Dokumentationsbögen) und kann durch DMP-Bögen, sofern diese verfügbar sind, unterstützt werden.

Der Patient wird zu Beginn der Studie durch den behandelnden Arzt mittels der Patienteninformation aufgeklärt. Es wird die schriftliche Patienteninformation eingeholt. Der Patient erhält die vollständige Patienteninformation in Kopie.

Danach bekommen die Patienten die Studienunterlagen ausgehändigt (mit entsprechenden Erklärungen). Bei Bedarf wird der Patient in die Durchführung des Stixens eingeführt. Während des ersten Besuches wird die Randomisierung durch das Koordinierungszentrum für klinische Studien Düsseldorf durchgeführt und der Patient der jeweiligen Behandlungsgruppe zugeordnet. Während des Besuches füllt der Arzt die CRF´s (ggf. mit Unterstützung der DMP-Bögen) aus. Bei den Patienten wird außerdem die Bestimmung des HbA1c durchgeführt. Ist bis zu 14 Tage vor Einschluss ein HbA1c bestimmt worden, so kann dieser verwendet werden. Der behandelnde Arzt erklärt dem Patienten die zugeordnete Therapie gemäß Studienprotokoll. Bei Nachfragen bezüglich des Handlings der Patiententagebücher und der Lebensqualitätsbögen steht die Dokumentationsassistentin des Koordinierungszentrum für klinische Studien Düsseldorf unter der Telefonnummer: 02 11/ 81 19 71 0 dem Arzt zur Verfügung.

Im Rahmen der Studie (oder des DMP-Programms) werden die Patienten nach einem viertel und einem halben Jahr erneut in die behandelnde Praxis/ das behandelnde Zentrum einbestellt. Zu diesen Terminen muss der behandelnde Arzt die CRF´s (ggf. mit Hilfe der DMP-Bögen) ausfüllen sowie die Patiententagebücher der Patienten einsammeln und den HbA1c-Wert bestimmen.

Die Patiententagebücher werden beim Monitoring pseudonymisiert und an das KKS gesandt. Der Patient bekommt zu jedem Studienbesuch ein neues Patiententagebuch ausgehändigt.

Bei Patienten bei denen keine ausreichende Dokumentation vorliegt, soll der Arzt eine verbesserte Dokumentation durch ein Gespräch erreichen. Bei Patienten, die nach eigenem Willen aus der Studie ausscheiden, soll der Ausschlussgrund eruiert werden. Nach sechs Monaten findet keine Führung mehr der Patienten bezüglich Blutzuckerselbstmessungen statt. Nach 12 Monaten wird der Patient erneut einbestellt und der HbA1c -Wert bestimmt.

### **12. Ermittlung der Wirksamkeit/ Erfassung der therapeutischen Effektivität**

### Fragen der Lebensqualität

Die Erhebung der gesundheitsbezogenen Lebensqualität und der Therapiecompliance und - Zufriedenheit erfolgt mittels Selbstbeurteilungsinstrumenten, die den Probanden während der Untersuchungen ausgehändigt und im DDFI vor Ort, bei den ambulanten Setting zu Hause ausgefüllt und dann an die Studienzentrale geschickt werden. In beiden Fällen muss sicher gestellt und dem Probanden mitgeteilt werden, dass der behandelnde Arzt keine Einsicht in die Bögen erhält, um einen Bias, insbesondere bei den Fragen zur Therapiezufriedenheit und Compliance zu vermeiden.

**12.1. Vergleich der gesundheitsbezogenen Lebensqualität:**

Die gesundheitsbezogene Lebensqualität (LQ), unter der im Allgemeinen die somatische, psychische und soziale Dimension von Krankheit verstanden wird, ist inzwischen ein etablierter Endpunkt bei der Beurteilung von Therapien (8). Eine Einführung dieses Maßes ist besonders bei Patienten mit chronischen Erkrankungen angezeigt, so auch bei Diabetespatienten (9). Bei dieser Patientengruppe zielt die Behandlung in der Regel nicht auf Heilung, sondern auf Kontrolle der chronischen Krankheit und der Vermeidung von Folgeschäden ab. Eine möglichst hohe gesundheitsbezogene Lebensqualität der Patienten ist daher ein wichtiges Ziel therapeutischen Handelns.

In dieser Studie soll durch eine Vorher-Nachher Messung überprüft werden, ob sich die Anzahl der Blutzuckerselbstmessungen auf die Lebensqualität der Probanden auswirkt. Eingesetzt werden zwei kurze und leicht verständliche Instrumente, da die Befragung insgesamt kurz gehalten werden soll, um den Aufwand für die Patienten zu minimieren und auf diese Weise Dropouts zu vermeiden.

Als globales Maß für die LQ wird der EuroQol-5D (5 Items) verwendet (10). Diese gut validierte Skala erlaubt die Bildung eines kontinuierlichen Scores und hat in Studien mit Diabetespatienten gut zwischen verschiedenen Therapiegruppen diskriminiert (11). Zudem eignet sich der EQ-5D besonders für gesundheitsökonomische Analysen (QALYS).

Um weiterhin krankheitsspezifischere Dimensionen der LQ abzufragen, werden zusätzlich Subskalen aus dem Profil der Lebensqualität chronisch Kranker erhoben. Die Skalen „physische Leistungsfähigkeit“ (8 Items) und psychisches Befinden (13 Items) werden gewählt, weil hier am ehesten Effekte der Blutzuckerselbstmessung zu erwarten sind (1). Der PLC ist für den Einsatz an chronisch Kranken validiert und bereits an Populationen mit Typ 2-Diabetes getestet worden.

Auf den Einsatz eines diabetesspezifischen Instruments wird verzichtet, weil in Frage kommende Instrumente entweder nicht in deutscher Übersetzung vorliegen (z.B. WHO Well-Being Q-28; ADDQoL), auf Typ I-Diabetiker zugeschnitten sind (z.B. IPC-Diabetes-Fragebogen) oder zu speziell erscheinen, als dass die hier getestete Intervention eine Veränderung erwarten lässt. Zudem werden mit dem DTSQ wichtige subjektive Faktoren bezüglich der Therapie des Diabetes erfasst (s. nächsten Abschnitt).

**12.2. Therapiezufriedenheit- und compliance**

Ein direkter Vergleich der Therapien erfolgt hinsichtlich der Zufriedenheit der Patienten und der Compliance mit der jeweiligen Therapie. Zur Messung der Therapiezufriedenheit steht mit dem Diabetes Treatment Satisfaction Questionnaire (DTSQ) ein validierter und in zahlreichen Studien eingesetzter Fragebogen zur Verfügung (12). Das Instrument erfragt neben der globalen Zufriedenheit auch die subjektiv wahrgenommene hyper- und hypoglykämische Kontrolle. Die Entwicklung der Zufriedenheit wird über die Zeitpunkte 0, 3 und 6 Monate beobachtet und zwischen den Gruppen verglichen.

Mit der Therapiecompliance ist die tatsächliche Umsetzung der Therapie durch den Patienten gemeint. Die Summary of Diabetes Self-Care Activities Measure (SDSCA) (13) fragt neben der studiengemäßen Blutzuckermessung auch nach anderen Aktivitäten der Selbsttherapie wie Medikation oder sportliche Betätigung. Dies erscheint einerseits wichtig, um die Angaben aus den Patiententagebüchern zu validieren und beurteilen zu können, ob sich die Patienten nicht auf eigene Kosten Kontrollinstrumente beschafft und diese angewendet haben. Andererseits soll auch untersucht werden, ob sich eine Reduktion der Selbstmessung auf andere diabetesbezogene Handlungsweisen auswirkt. Zudem gibt es Hinweise darauf, dass der Umfang der selbsttherapeutischen Handlungen die kognitive Präsenz des „krankseins“ verstärkt und darüber zu einer schlechteren subjektiven Lebensqualität führen kann (14). Der SDSCA wird aus Praktikabilitätsgründen in der Studie in einer verkürzten Version eingesetzt.

**12.3. Gesundheitsökonomische Betrachtung**

Die vorliegende Studie ermöglicht die ökonomische Untersuchung der Kosten der Blutzuckermessung und des gesundheitlichen Ergebnisses (Outcome). Das pragmatische Studiendesign ermöglicht einerseits eine Einschätzung der entstehenden Kosten in einem realistischen Umfeld, und andererseits eine breite Anwendung der Ergebnisse. Diese Anwendbarkeit wird durch die Wahl von zwei sehr unterschiedlichen Settings a) Hausarztpraxen und b) ein tertiäres Versorgungszentrum ermöglicht. Die Cost-Effectiveness Analyse geschieht bei erwartetem äquivalenten Stoffwechselergebnis als Cost Minimisation Analysis unter Einbeziehung der Lebensqualität der Patienten. Durch die zwei verschiedenen Settings ergibt sich unserer Ansicht nach keine Notwendigkeit für ein extensives Modelling, um eine Übertragung der Ergebnisse zu ermöglichen (15).

In Anbetracht des Verlaufes des Diabetes geschieht die Auswertung der gesundheitsökonomischen Ergebnisse hierachisch. Primär ist für die ökonomische Auswertung die Äquivalenz der Therapien bezüglich der Langzeitfolgen relevant. Nur wenn diese Äquivalenz gegeben ist (Intention-to-treat Analyse), so scheint es sinnvoll weitergehende Testungen durchzuführen. Dies schliesst die Analyse der gefährlichen Hyper- und Hypoglykaemien mit ein.

Im zweiten Schritt wird die Lebensqualität der Patienten verglichen. Wird hier keine Äquivalenz oder Überlegenheit der Gruppe mit der selteneren Blutzuckermessung erreicht, so muss die weitere Analyse hinterfragt werden.

Im dritten Schritt werden dann die direkten Kosten für die Blutzuckermessung verglichen.

Im letzten Schritt geschieht die explorative Auswertung bezüglich eventueller Änderungen des Gesundheitsverhaltens im Vergleich zwischen beiden Gruppen. Sollten sich hier überraschend gravierende Unterschiede bezüglich der entstehenden Kosten ergeben, so müssen diese Ergebnisse vorsichtig diskutiert werden.

**12.3.1. Folgekosten**

Die gesundheitsökonomische Betrachtung lässt sich bei einer chronischen Erkrankung mit lebensbedrohlichen Langzeitfolgen nicht von den klinischen Ergebnissen trennen.

Gesundheitsökonomisch fallen bei einer chronischen Erkrankung, wie dem Typ 2- Diabetes, die krankheitsbezogenen Kosten v.a. in der Versorgung der Spätfolgen an, die von der optimalen Stoffwechseleinstellung abhängig sind. Der HbA1c Wert, der diese Stoffwechseleinstellung widerspiegelt, ist damit auch für eine gesundheitsökonomische Analyse der wichtigste Zielparameter. Sind die beiden Studiengruppen bezüglich der Stoffwechseleinstellung äquivalent, so sind keine langfristigen gesundheitsökonomischen Folgekosten einer Umstellung auf ein weniger intensives Selbstmessungsregime zu erwarten. Eine Gefährdung der Patienten durch Hypo- oder Hyperglykaemien muss dabei ausgeschlossen werden.

**12.3.2. Direkte Kosten der Blutzuckermessung**

Sollte sich die Stoffwechseleinstellung zwischen beiden Gruppen nicht unterscheiden, so ist damit für die gesundheitsökonomische Untersuchung nur die Analyse der eingesparten Kosten für die BZ Messung relevant, da vermehrte Spätschäden nicht auftreten.

Die Kosten für die Blutzuckermessung müssen damit genau erhoben werden, da die zu erwartende Reduktion der Kosten durch eine Verminderung der Messfrequenz abgeschätzt werden muss und nicht auf einfachen Modellen basierend auf ärztlichen Empfehlungen abgeschätzt werden kann. Diese Kostenreduktion ist primär von der tatsächlich durchgeführten Zahl der Selbstmessungen abhängig. Die Blutzuckermessungen werden auch schon bisher von den Patienten erfasst und im Rahmen der Studie analysiert und zwischen beiden Gruppen verglichen. Dieses Vorgehen hat den Vorteil, dass es keinerlei zusätzliche Dokumentation notwendig macht und damit weder die behandelnden Ärzte noch die Patienten belastet.

**12.3.3. Erfassung möglicher indirekter Kosten**

Es ist möglich, allerdings gibt es dafür keinen Anhalt, dass eine verminderte Frequenz bei der Selbstmessung des BZ zu einer Veränderung des Patientenverhaltens führt. Daher wird das Krankheitsverhalten der Patienten im Rahmen der Studie genau beobachtet. Es werden das Krankheitsverhalten der Patienten betreffende ökonomische Daten erhoben, die einen Hinweis auf eine solche Entwicklung geben könnten.

Primär steht ein Vergleich der entstehenden direkten Kosten zwischen beiden Gruppen im Vordergrund und nicht die Quantifizierung der Gesamtkosten. Deshalb wird auch keine Detailerhebung durchgeführt, die bei den zur Verfügung gestellten Mitteln nicht ökonomisch machbar wäre. Erhoben werden für beide Gruppen 1. Arztbesuche, unabhängig von den Disziplinen, 2. Zahl der Krankenhausaufenthalte und 3. Zahl der Tage mit Arbeitsunfähigkeit. Bei der Analyse wird dabei davon ausgegangen, dass die Gruppen bei Studieneinschluss sich lediglich in Ihrer Messfrequenz und nicht bezüglich ihrer Stoffwechseleinstellung unterscheiden.

#### 13. Ermittlung der Sicherheit

**13.1. Erfassung und Bewertung von unerwünschten Ereignissen**

Begriffsbestimmungen

**13.1.1. Unerwünschte Ereignisse**

Unerwünschte Ereignisse sind Erkrankungen, Krankheitszeichen oder Symptome, die nach Einschluß des Patienten in die Studie eintreten oder sich verschlechtern.

Die Ausprägung wird wie folgt bewertet:

- gering
- mäßig
- schwer
- lebensbedrohend

Für jedes Ereignis ist eine Kausalitätsbewertung vorzunehmen:

- kein Zusammenhang
- möglicher Zusammenhang
- wahrscheinlicher Zusammenhang
- sicherer Zusammenhang

**13.1.2. Schwerwiegende unerwünschte Ereignisse**

Unerwünschte Ereignisse werden in folgenden Fällen als schwerwiegend definiert:

Jeder Todesfall, unabhängig von der Todesursache, der innerhalb von 30 Tagen nach Abschluss der protokollgemäßen Therapie auftritt.

- Lebensbedrohliche /-bedrohende Erkrankungen
- Ereignisse. die zu einer permanenten Behinderung führen
- Ereignisse, die eine Hospitalisierung erfordern oder einen Krankenhausaufenthalt verlängern
- Überdosierung, die zu Symptomen führt
- Die Entwicklung eines Medikamentenmissbrauchs oder einer Medikamentenabhängigkeit
- Hypoglykämie mit Fremdhilfe und hyperosmolares Koma

**13.2. Dokumentation und Bewertung unerwünschter Ereignisse**

Für die Erfassung der UE/SUE steht in den Zentren ein Dokumentationsbogen zur Verfügung. Bei den Arztpraxen erfolgt die Dokumentation der UE in einem separaten Bogen mit Unterstützung der DMP-Bogen. Ein SUE wird gesondert auf einem Dokumentationsbogen erfasst.

Dokumentation und Meldung schwerwiegender oder unerwarteter unerwünschter Ereignisse erfolgt von den Zentren/Praxen schnellstmöglich (innerhalb von 24 Stunden nach Bekannt werden) an den Studienleiter oder an die Studienzentrale.

| Studienleiter:  Prof. Dr. med. W. A. Scherbaum  Deutsches Diabetes Zentrum an der Heinrich-Heine-Universität Düsseldorf,  Deutsche Diabetes Klinik  Auf'm Hennekamp 65  40225 Düsseldorf  Tel.: 0211 / 33 82 200  Telefax: 0211 / 33 69 103,  e-mail: scherbaum@ddfi.uni-duesseldorf.de | Studienzentrale:  Prof. Dr. rer. nat. C. Ohmann, Koordinierungszentrum für Klinische Studien an der Heinrich-Heine-Universität,  z.Hd. Frau Janett Schindler  Moorenstr.  5, 40225 Düsseldorf,  Telefon: 02211 / 811 9710  Telefax: 0211 / 8119705,  e-mail: janett.schindler@uni-duesseldorf.de |
| --- | --- |

Für jedes dieser Ereignisse ist ein Berichtsbogen auszufüllen und umgehend (innerhalb von 7 Tagen) an, die angegebene Adresse weiterzuleiten. Wenn die erforderlichen Informationen initial nicht verfügbar sind, müssen Folgeberichte abgefasst werden. Bei Todesfällen sollte nach Möglichkeit eine Kopie des Autopsieberichts beigelegt werden.

Die Studienleiter werden die zuständige Ethikkommission über die gemeldeten schwerwiegenden und unerwarteten unerwünschten Ereignisse in Kenntnis setzen, sofern diese sicher oder wahrscheinlich kausal mit der Studienintervention in Verbindung stehen.

Die an der Studie beteiligten Prüfzentren/-ärzte werden von der Studienleitung über alle gemeldeten Ereignisse in regelmäßigen Abständen informiert. Sollten sich aus den unerwünschten Ereignissen Veränderungen im therapeutischen Vorgehen ergeben, so erfolgt diese Information sofort.

**14. Dauer der Studie / Studienabbruch**

**14.1. Reguläres Studienende**

Die Studie hat eine Gesamtdauer von ca. 24 Monaten. Davon entfallen 6 Monate auf die Rekrutierung.. Der Follow-Up Zeitraum umfasst 12 Monate. Für die Datenanalyse und das Erstellen des Abschlussberichts sind weitere 6 Monate vorgesehen.

**14.2. Vorzeitiges Studienende / Abbruch der gesamten Studie**

Als Gründe für einen vorzeitigen Abbruch der gesamten Studie kommen in Frage:

- Entscheidung der Studienleitung bei unvertretbaren Risiken und Toxizitäten unter Nutzen-Risiko-Abwägung
- neue (wissenschaftliche) Erkenntnisse während der Laufzeit der Studie,
- nicht adäquate Rekrutierungsrate.

Entscheidungsgremium

Die Entscheidung über den vorzeitigen Abbruch der Studie wird von den Studienleitern getroffen.

**15. Statistische Methoden**

In die Studie aufgenommen werden Frauen und Männer im Alter von 35 bis 80 Jahren, die eine Insulintherapie oder eine Behandlung mit oralen Antidiabetika bekommen.

**15.1. Grundlage der Berechnung**

Für dieses Patientenkollektiv wurde auf der Basis von Daten des Deutschen Diabetes Forschungsinstituts Düsseldorf eine Auswertung über die statistische Verteilung des Hauptzielkriteriums der Studie, dem HbA1c-Wert durchgeführt (MW = 9,95; SD plus/minus 1,83; N = 590). Diese Daten dienten dann als Grundlage für die Fallzahlberechnung.

**15.2. Studienhypothesen**

Die Auswertung der Studie geschieht für beide Settings getrennt bei folgender Hypothese:

H_0_: Die hochfrequente Blutzuckermessung ist der einmal wöchtlichen Blutzuckermessung bezüglich der Entwicklung des Hb A1c Wertes überlegen.

H_1_: Eine Überlegenheit der hochfrequenten Blutzuckermessung bezüglich des HbA1c Wertes lässt sich nicht nachweisen.

**15.3. Auswertungsfolge**

Sollte H_0_ für beide Settings verworfen werden können, erfolgt im nächsten Schritt die gepoolte Analyse aller Patienten. Daran anschließend werden die explorativen Subgruppenanalysen der sekundären Zielparameter durchgeführt (s.o.).

**15.4. Intention-to-treat / Per-protocol Analyse**

Für den hier vertretenen Ansatz einer Studie zur Versorgungsforschung, die die primäre Aufgabe, hat die aktuelle Situation der Therapie darzustellen und mit einem anderen Vorgehen zu kontrastieren, erscheint uns eine intention-to-treat Analyse als die sinnvollste Form der Auswertung. Sollte eine große Zahl von Patienten nicht in der Lage oder willens sein, eine regelmäßige Kontrolle des Blutzuckers durchzuführen, so bildet die Intention-to-treat Analyse dieses Verhalten am unverzerrtesten ab.

Im Gegensatz zur einer Überlegenheitstestung ist die intention-to-treat Analyse allerdings für eine Äquivalenzstudie die weniger konservative Herangehensweise, da sich die beiden Strategien der Verlaufskontrolle möglicherweise angleichen. Dies kann einerseits durch eine Non-Compliance in der hochfrequenten Gruppe oder möglicherweise durch vermehrte Messungen in der niedrigfrequenten Gruppe geschehen, beides würde die Unterschiede zwischen den Gruppen reduzieren. Aus diesem Grunde werden alle Analysen explorativ ebenso per-protocol durchgeführt. In diese Per-protocol-Analyse werden alle Patienten aufgenommen, die in der hochfrequenten Gruppe mehr als 75% der vom Arzt empfohlenen Messungen durchführen und in ihren Tagebüchern dokumentieren. Für die niedrigfrequente Gruppe gilt zusätzlich, dass die empfohlenen Messungen nicht um mehr als 10% überschritten werden sollten.

**15.5. Fallzahlkalkulation**

Als statistische Methode der Hypothesenprüfung soll ein t-Test auf Äquivalenz durchgeführt werden. Dabei wird ein Unterschied des HbA1c-Wertes von 0,5% als klinisch signifikant angenommen. Bei einem Alpha-Fehler von 0,05 und einer Power von 80% im einseitigen t-Test ist eine Fallzahl von 167 Personen pro Studienarm nötig, um bei den angenommenen Kennwerten eine Äquivalenz nachzuweisen.

Da die konfirmatorischen Analysen nach dem Intention-to-treat-Ansatz durchgeführt werden sollen, rechnen wir mit einem geringen Drop- out, etwa durch Rücknahme der Einverständniserklärung. Daher sollten insgesamt 100 Patienten pro Studienarm eingeschlossen werden. Bei zwei Studienarmen (Insulin/Oral) für beide Therapiegruppen (Min/Max) ergibt sich eine Gesamtfallzahl von 400 Patienten.

**15.6. Randomisierung**

Die Randomisation erfolgt stratifiziert nach Insulin oder oraler Antidiabetikertherapie und nach Zentren in permutierter Blockrandomisation. Die Blöcke sind zwischen 4 und 8 Patienten groß. Die Erstellung der Listen geschieht mit dem Programm Rand von IDV, Garching bei München.

#### 16. Datenmanagement

#### 16.1. Patientenidentifikationsliste

Alle patientenbezogenen Daten werden in pseudonymisierter Form im Koordinationszentrum für Klinische Studien erfasst. Jeder Patient wird mit Initialen, Geburtsjahr und einer Patientennummer, die bei der Registrierung zugewiesen wird, unverwechselbar gekennzeichnet. Der Prüfarzt führt eine vertrauliche Patientenliste, in dem die Patientennummer mit dem vollen Patientennamen verbunden sind.

#### 16.2. Datenerhebung / Dokumentationsbögen

Die Datenerhebung erfolgt anhand von Dokumentationsbögen (Case Report Forms), die von der Studienleitung zur Verfügung gestellt werden. Ein Muster aller Bögen befindet sich im Anhang. Die CRF´s liegen als Original beim Studienarzt vor. Die Studienzentrale erhält eine Kopie.

Die Bögen sind mit Tinte oder Kugelschreiber auszufüllen, Bleistifteintragungen sind nicht erlaubt. Korrekturen sind wie folgt vorzunehmen: Der falsche Eintrag wird mit einer einfachen Linie durchgestrichen, die korrekte Information daneben eingetragen und vom Prüfarzt mit Datum paraphiert und ggf. mit Angabe des Grundes der Korrektur versehen. Datenfelder, die wegen fehlender Information nicht ausgefüllt werden können, sind zu kommentieren.

Die Bögen sind zeitnah auszufüllen und anschließend vom Prüfarzt zu kontrollieren, mit Datum zu unterschreiben und eine Kopie an die Studienzentrale zu schicken.

Optional können die Daten direkt über moderne Softwareprodukte erfasst werden, die es den Ärzten erlauben, die Daten über einen Internet-Browser direkt in die Datenbank einzugeben. Die in den elektronischen Dokumentationsbögen erfassten Daten enthalten ebenfalls nur die Initialen, das Geburtsjahr und die Patientennummer. Bei der Datenverarbeitung werden besondere Maßnahmen des Datenschutzes und der Datensicherheit ( z.B. Kryptographie) eingehalten.

Für die Datenerhebung in den Arztpraxen können die DMP-Bögen herangezogen werden, vorausgesetzt, sie werden von der Kassenärztlichen Vereinigung zur Verfügung gestellt. Die Regeln zur Datenerhebung der DMP- Bögen sind den Ärzten durch Schulungen hinreichend bekannt. Eine Anleitung zum Ausfüllen ist auf der Homepage der KV ersichtlich.

#### 16.3. Datenverarbeitung

Das zentrale Datenmanagement erfolgt im Koordinationszentrum für Klinische Studien, Universität Düsseldorf, nach den Richtlinien von GCP und zentrumsinternen Standard Operating Procedures.

Die Dateneingabe mit Plausibilitätsabfragen erfolgt über eine spezielle Studiensoftware.

Die Überprüfung der Richtigkeit der Daten erfolgt durch Range-, Validitäts- und Konsistenzchecks der für die Studie parametrierten und konfigurierten Software, sowie durch eine visuelle Kontrolle vor der Eingabe. Nicht plausible oder fehlende Daten werden als Querys dokumentiert und an den Monitor zur Auflösung bei den Studienärzten weitergeleitet. Nach Auflösung von Queries werden die Korrekturbelege zusammen mit den Dokumentationsbögen aufbewahrt.

Die Ablage der Daten erfolgt auf einem Firewall geschützten Oracle Datenbank-Server mit regelmäßigem Backup.

Am Studienende wird nach Eingabe aller Eintragungen und der Aufbereitung der Daten zur Analyse die Datenbank geschlossen. Dieser Vorgang wird dokumentiert.

#### 16.4. Aufbewahrung der Studienunterlagen

Die Originale aller zentralen Studiendokumente einschließlich Dokumentationsbögen werden in der Studienzentrale für mindestens 15 Jahre nach Erstellung des Abschlußberichts aufbewahrt.

Alle Queries und sonstige Korrekturen sind an die Studienzentrale zu übergeben, wo sie zusammen mit den Dokumentationsbögen archiviert werden.

Der Studienarzt bewahrt die angefallenen administrativen Dokumente (Schriftverkehr mit Ethikkommission, Überwachungsbehörde, Studienleitung, Studienzentrale etc.), die Patientenidentifikationsliste, die unterschriebenen Einwilligungserklärungen, Kopien der Dokumentationsbögen und der allgemeinen Studiendokumentation (Protokoll, Amendments) für die oben genannte Zeit auf.

Originaldaten der Studienpatienten (Krankenakten) sind entsprechend der für die Prüfzentren gültigen Archivierungsfrist aufzubewahren.

#### 17. Qualitätssicherung

#### 17.1.Standardisierung

#### Die Bestimmung des HbA1c-Wertes wird in den Laboren der jeweiligen Zentren bzw. den angegliederten Hausarztpraxen durchgeführt. Die Labore der Hausarztpraxen sind durch ihre Teilnahme am DMPG – Programm „Diabetes“ bei der Kassenärztlichen Vereinigung

#### 17.2. Kontrolle des Studienablaufs und der Datenqualität

Es ist vorgesehen, ein Monitoringprogramm zu implementieren. Eine vollständige Source Data Verification findet jedoch aus Kostengründen nicht statt. Pro Zentrum wird vor Beginn der Studie ein Initierungsbesuch durchgeführt.

Die Frequenz der regulären Monitoringbesuche muss am Budget orientiert werden. Ein ein- oder am besten zweimaliger Besuch ist jedoch auf jeden Fall nötig. Ein Zugang des Monitors zu den Studienunterlagen sollte in der Prüfvereinbarung und durch entsprechende Passagen in der Patienteninformation und zugesichert werden.

Für den Studienablauf innerhalb der verschiedenen Zentren gelten verschiedene Qualitätsindikatoren, die auch für die Gesamtstudie Gültigkeit haben. Ein zeitgerechter Ablauf der Studie ist Grundvoraussetzung für die Erreichung praktikabler Ergebnisse, dazu gehört die Überprüfung der Rekrutierungsrate als essentielle Vorraussetzung für die erfolgreiche Durchführung der Studie. Die Erfassung unerwünschter Ereignisse, des HbA1c-wertes, Änderungen der Diabetestherapie und Daten zur Lebensqualität und Gesundheitsökonomie sind ohne Einhaltung der Untersuchungs- und Bewertungstermine nicht möglich. Dieser Aspekt muss spezieller Kontrolle unterliegen.

#### 18. Ethische Grundlagen

Das Studienprotokoll wird bei den zuständigen Ethikkommissionen eingereicht. Die Patienten sind in enger ärztlicher Betreuung und ihre Stoffwechseleinstellung wird regelmäßig monitoriert, daher besteht jederzeit die Möglichkeit, die Frequenz der Blutzuckermessungen den Bedürfnissen der Patienten anzupassen, ohne dass Risiken für das Wohlbefinden der Patienten entstehen oder die Aussage der Studie gefährdet wird. Die Durchführung der Studie geschieht nach den Vorgaben der Deklaration von Helsinki, 1996.

**19.Lit****eratur**

[1] Franciosi M, Pellegrini F, De Berardis G, Belfiglio M, Cavaliere D, Di Nardo B et al. The impact of blood glucose self-monitoring on metabolic control and quality of life in type 2 diabetic patients: an urgent need for better educational strategies. Diabetes Care 2001; 24(11):1870-1877.

[2] Greenfield S, Kaplan SH, Silliman RA, Sullivan L, Manning W, D'Agostino R et al. The uses of outcomes research for medical effectiveness, quality of care, and reimbursement in type II diabetes. Diabetes Care 1994; 17 Suppl 1:32-39.

[3] Harris MI. Frequency of blood glucose monitoring in relation to glycemic control in patients with type 2 diabetes. Diabetes Care 2001; 24(6):979-982.

[4] Scorpiglione N, el Shazly M, Abdel-Fattah M, Belfiglio M, Cavaliere D, Carinci F et al. Epidemiology and determinants of blood glucose self-monitoring in clinical practice. Diabetes Res Clin Pract 1996; 34(2):115-125.

[5] Faas A, Schellevis FG, Van Eijk JT. The efficacy of self-monitoring of blood glucose in NIDDM subjects. A criteria-based literature review. Diabetes Care 1997; 20(9):1482-1486.

[6] Coster S, Gulliford MC, Seed PT, Powrie JK, Swaminathan R. Monitoring blood glucose control in diabetes mellitus: a systematic review. Health Technol Assess 2000; 4(12):i-93.

[7] http://www.riedborn-apotheke.de/ letzter Zugriff 27-8-2002

[8] Sanders C, Egger M, Donovan J, Tallon D, Frankel S. Reporting on quality of life in randomised controlled trials: bibliographic study. BMJ 1998; 317(7167):1191-1194.

[9] Bradley C. Importance of differentiating health status from quality of life. Lancet 2001; 357(9249):7-8.

[10] EuroQol--a new facility for the measurement of health-related quality of life. The EuroQol Group. Health Policy 1990; 16(3):199-208.

[11] Redekop WK, Koopmanschap MA, Stolk RP, Rutten GE, Wolffenbuttel BH, Niessen LW. Health-related quality of life and treatment satisfaction in Dutch patients with type 2 diabetes. Diabetes Care 2002; 25(3):458-463.

[12] Bradley C. Diabetes treatment satisfaction questionnaire. Change version for use alongside status version provides appropriate solution where ceiling effects occur. Diabetes Care 1999; 22(3):530-532.

[13] Toobert DJ, Hampson SE, Glasgow RE. The summary of diabetes self-care activities measure: results from 7 studies and a revised scale. Diabetes Care 2000; 23(7):943-950.

[14] Watkins KW, Connell CM, Fitzgerald JT, Klem L, Hickey T, Ingersoll-Dayton B. Effect of adults' self-regulation of diabetes on quality-of-life outcomes. Diabetes Care 2000; 23(10):1511-1515.

[15] Drummond MF, Jefferson TO. Guidelines for authors and peer reviewers of economic submissions to the BMJ. The BMJ Economic Evaluation Working Party. BMJ 1996; 313(7052):275-283.
