# Supplementary material for: Effect of the Frequency of Self-Monitoring Blood Glucose in Patients with Type 2 Diabetes Treated with Oral Antidiabetic Drugs—A Multi-Centre, Randomized Controlled Trial
Source: PLoS One. 2008 Aug 28;3(8):e3087. doi: 10.1371/journal.pone.0003087 (PMC2518209; doi:10.1371/journal.pone.0003087)
Supplement: Protocol S2 — Trial Protocol, in English. (0.02 MB PDF) [file pone.0003087.s002.pdf]

Written for Controlled-Trials.com: http://www.controlled-trials.com/ISRCTN79164268

Evaluation of the necessary frequency of blood glucose self-monitoring in type 2 diabetic patients. A prospective, controlled, randomised, multicenter study.

**ISRCTN**	ISRCTN79164268
**ClinicalTrials.gov identifier**	
**Public title**	Evaluation of the necessary frequency of blood glucose self-monitoring in type 2 diabetic patients. A prospective, controlled, randomised, multicenter study.
**Scientific title**	
**Acronym**	N/A
**Serial number at source**	N/A
**Study hypothesis**	To evaluate the impact of the frequency of blood glucose self-monitoring on glycaemic control (HbA1c and occurrence of hypoglycaemia) of type 2 diabetic patients. Currently there are no general recommendations on the frequency of self blood glucose monitoring in type 2 diabetics treated with a fixed insulin regime or oral antidiabetic medication. The study intends to compare over a follow-up period of 6 month patients with a high frequency of self-monitoring with those with a low frequency. This comparison is done separately for two groups of patients:<br>1. Treated with a fixed insulin regime<br>2. Treated with oral antidiabetic drugs only<br>Satisfaction with the recommended treatment is a secondary endpoint of the study.
**Ethics approval**	Not provided at time of registration
**Study design**	Randomised controlled trial
**Countries of recruitment**	Germany
**Disease/condition/study domain**	Type 2 diabetes mellitus
**Participants - inclusion criteria**	1. Patients treated with a fixed dose of mixture insulin twice a day<br>2. Patients who are treated with one or more oral antidiabetic drugs<br>3. From 35 to 80 years<br>4. Informed consent<br>5. Type 2 diabetic patients
**Participants - exclusion criteria**	1. Treatment with multiple insulin injections (more than 2/day)<br>2. Type 1 diabetic patients<br>3. Advanced renal insufficiency (known creatinine >2.5 mg/dl)<br>4. >2 hypoglycaemia with necessary outside help within the last three months<br>5. Hypoglycaemic shock/hyperosmolaric coma within the last three months<br>6. Pregnancy<br>7. Severe impaired vision<br>8. Communication problems<br>9. Home care/nursing service

**Anticipated start date**	01/12/2003
**Anticipated end date**	01/10/2006
**Status of trial**	Completed
**Patient information material**	
**Target number of participants**	400 (100 in each group)
**Interventions**	Blood glucose monitoring<br><br>1. Patients on insulin mixture:<br>1.1 Maximum recommendation: every day fasting, every second day before dinner, additional once a profile<br>1.2 Minimum recommendation: once a week a fasting blood glucose<br><br>2. Patients who get an oral antidiabetic:<br>2.1 Maximum recommendation: every second day a fasting blood glucose, once a week a blood sugar check before dinner<br>2.2 Minimum recommendation: one fasting blood glucose per week
**Primary outcome measure(s)**	HbA1c six months after start of the study
**Secondary outcome measure(s)**	1. Hypoglycaemia with necessary outside help<br>2. Hyperosmolar coma<br>3. Quality of life<br>4. Compliance<br>5. HbA1c after 12 months
**Sources of funding**	Federal Ministry of Education and Research (Bundesministerium Für Bildung und Forschung [BMBF]) - Germany - 01GL0303
**Trial website**	
**Publications**	
**Contact name**	**Prof Werner A. Scherbaum**
Address	Deutsches Diabetes Zentrum an der Heinrich-Heine-Universität<br>Auf´m Hennekamp 65
City/town	Duesseldorf
Zip/Postcode	40225
Country	Germany
Tel	+49 (0)2113382-200
Fax	+49 (0)2113369103
Email	scherbaum@ddz.uni-duesseldorf.de
**Sponsor**	Federal Ministry of Education and Research (Bundesministerium Für Bildung und Forschung [BMBF]) (Germany)
Address	Südstrasse 125
City/town	Bonn

Zip/Postcode	53175
Country	Germany
Tel	+49 (0)2283821202
Fax	+49 (0)2283821257
Email	cornelia.bormann@dlr.de
**Date applied**	05/09/2005
**Last edited**	18/02/2008
**Date ISRCTN assigned**	28/10/2005
